# Dysregulation of Long Non-coding RNAs and mRNAs in Plasma of Clear Cell Renal Cell Carcinoma Patients Using Microarray and Bioinformatic Analysis

**DOI:** 10.3389/fonc.2020.559730

**Published:** 2020-11-27

**Authors:** Bing Zhang, Wei Chu, Feifei Wen, Li Zhang, Lixia Sun, Baoguang Hu, Jingjing Wang, Qingguo Su, Yanhui Mei, Jingyuan Cao, Jing Zheng, Xiaodong Mou, Hongliang Dong, Xiaoyan Lin, Nan Wang, Hong Ji

**Affiliations:** ^1^Department of Urology, Binzhou Medical University Hospital, Binzhou Medical University, Binzhou, China; ^2^Department of Pathology, Binzhou Medical University Hospital, Binzhou Medical University, Binzhou, China; ^3^Department of Anesthesiology, Binzhou Medical University Hospital, Binzhou Medical University, Binzhou, China; ^4^Department of Gastrointestinal Surgery, Binzhou Medical University Hospital, Binzhou Medical University, Binzhou, China; ^5^Clinical Medicine Laboratory, Binzhou Medical University Hospital, Binzhou Medical University, Binzhou, China; ^6^Department of Pathology, Shandong Province Hospital, Jinan, China

**Keywords:** clear cell renal cell carcinoma, bioinformatics, long non-coding RNA, mRNA, microarray, differentially expressed gene

## Abstract

**Objective:** The roles of long non-coding RNAs (lncRNAs) in the diagnosis of clear cell renal cell carcinoma (ccRCC) are still not well-defined. We aimed to identify differentially expressed lncRNAs and mRNAs in plasma of ccRCC patients and health controls systematically.

**Methods:** Expression profile of plasma lncRNAs and mRNAs in ccRCC patients and healthy controls was analyzed based on microarray assay. Gene ontology (GO) and Kyoto Encyclopedia of Genes and Genomes (KEGG) pathway-based approaches were used to investigate biological function and signaling pathways mediated by the differentially expressed mRNAs. SOCS2-AS1 was selected for validation using Real-Time PCR. The differentially expressed lncRNAs and mRNAs were further compared with E-MTAB-1830 datasets using Venn and the NetworkAnalyst website. The GEPIA and ULCAN websites were utilized for the evaluation of the expression level of differentially expressed mRNA and their association with overall survival (OS).

**Results:** A total of 3,664 differentially expressed lncRNAs were identified in the plasma of ccRCC patients, including 1,511 up-regulated and 2,153 down-regulated lncRNAs (fold change ≥2 and *P* < 0.05), respectively. There were 2,268 differentially expressed mRNAs, including 932 up-regulated mRNAs and 1,336 down-regulated mRNAs, respectively (fold change ≥2 and *P* < 0.05). Pathway analysis based on deregulated mRNAs was mainly involved in melanogenesis and Hippo signaling pathway (*P* < 0.05). In line with the lncRNA microarray findings, the SOCS2-AS1 was down-regulated in ccRCC plasma and tissues, as well as in cell lines. Compared with the E-MTAB-1830 gene expression profiles, we identified 18 lncRNAs and 87 mRNAs differently expressed in both plasma and neoplastic tissues of ccRCC. The expression of 10 mRNAs (*EPB41L4B, CCND1, GGT1, CGNL1, CYSLTR1, PLAUR, UGT3A1, PROM2, MUC12*, and *PCK1*) was correlated with the overall survival (OS) rate in ccRCC patients based on the GEPIA and ULCAN websites.

**Conclusions:** We firstly reported differentially expressed lncRNAs in ccRCC patients and healthy controls systemically. Several differentially expressed lncRNAs and mRNAs were identified, which might serve as diagnostic or prognostic markers. The biological function of these lncRNAs and mRNAs should be further validated. Our study may contribute to the future treatment of ccRCC and provide novel insights into cancer biology.

## Backgrounds

Renal cell carcinoma (RCC) is one of the most lethal urological cancers with an incidence of ~4% in adults (1–3). There are several histological subtypes of RCC, among which clear-cell RCC (ccRCC) is the most common type accounting for 65–70% of all renal malignancies (4). It originated from the epithelial cells of the proximal renal tubule and was associated with high possibility of metastasis and poor prognosis. To date, the treatment of metastatic RCC is still a challenge as most of the ccRCC cells present resistance to chemotherapy and radiotherapy (1). Recently, anti-angiogenic drugs targeting vascular endothelial growth factor (VEGF) signaling, such as multiple kinase inhibitors (e.g., Sunitinib or Pazopanib), have been approved for treating patients with advanced or metastatic ccRCC. However, the therapeutic effects are limited to only a short time, with many patients presenting relapse eventually (5, 6). Therefore, early diagnosis and management together with regular follow-up are essential. Biomarkers from the circulating system (e.g., plasma and serum) provide a convenient and non-invasive method for the diagnosis of a tumor. However, the utility of circulating tumor-specific biomarkers, such as carbohydrate antigen-125 (CA-125) and carcinoembryonic antigen (CEA), is still limited in the diagnosis and follow-up of ccRCC. On this basis, it is essential to identify new blood biomarkers for ccRCC.

Non-coding RNAs (ncRNAs) in plasma or serum, such as mircroRNA and long non-coding RNAs (lncRNAs), are considered as novel non-invasive biomarkers for cancers (7–9). LncRNAs are conventionally defined as a transcript longer than 200 nucleotides (nt) in length with no protein-coding capability. They are stable in tissues and body fluids including urine and blood, preventing from endogenous RNase degradation. To our best knowledge, plasma lncRNA signatures are related to the pathogenesis of a variety of neoplasms, including prostate cancer (10), breast cancer (11), gastric cancer (9, 12), as well as esophageal squamous cell carcinoma (13).

Over the past decades, several studies had focused on the expression profile of lncRNA in ccRCC tissues (14–23). Many lncRNAs are confirmed to be involved in the pathogenesis of ccRCC (19, 21, 23, 24). Nevertheless, few studies have evaluated the roles of plasma lncRNAs in the diagnosis of ccRCC. For example, Wu et al. reported a risk model consisting of five serum lncRNAs (i.e., lncRNA-LET, PVT1, PANDAR, PTENP1, and linc00963) that could distinguish the benign renal tumors from ccRCC (25). Therefore, the aim of the present study was to systematically explore potential plasma-derived lncRNA biomarkers for early diagnosis and follow-up of ccRCC.

## Materials and Methods

### Patients and Samples Processing

This study was approved by the Medical Ethics Committee of Binzhou Medical University Hospital (approval No. LW-017). All participants signed the written informed consent.

Plasma samples were obtained from 29 ccRCC patients admitted to our hospital between December 2016 and February 2019. None of the patients received any treatment before enrolling into this study. Twenty-four volunteers without any urological system disorders served as controls. Whole blood was collected from each participant and then was stored in EDTA tubes as an anticoagulant followed by centrifugation at 1,000 g for 10 min at 4°C. Plasma was then transferred to sterile polypropylene tubes on ice and centrifuged again at 10,000 g for 10 min at 4°C to remove cell debris. The supernatant was then aliquoted into 800 μL per tube and stored in liquid nitrogen until further analysis.

Twenty-seven ccRCC cancerous tissues were collected, together with 18 paired adjacent non-tumorous specimens. None of the patients received any anti-tumor treatment prior to surgery. Samples from tumor and normal renal tissues were immediately frozen in liquid nitrogen overnight and stored at −80°C for further analysis. Hematoxylin and eosin (H&E) stained sections were utilized to determine the histological features. The final histological diagnosis was made with the formalin-fixed paraffin-embedded tissue samples. Histological changes were reviewed by two experienced pathologists blinded to this study.

### Cell Culture

The ccRCC cell lines (i.e., ACHN and 786-O) were purchased from the Institute of Biochemistry and Cell Biology (Shanghai, China). The HK-2 human kidney tubular epithelial cell line was purchased from the American Type Culture Collextios (Manassas, VA, USA). ACHN cells were cultured in Eagle's Minimum Essential Medium supplemented with 10% fetal bovine serum (FBS). The 786-O cells were cultured in RPMI-1640 medium supplemented with 10% FBS (Gibco; Thermo Fisher, Waltham, MA, USA). The HK-2 cells were cultured in KSF medium (Gibco; Thermo Fisher, Waltham, MA, USA) containing epidermal growth factor (PeproTech, Rocky Hill, NJ, USA). All cells were cultured at 37°C in a humidified incubator with 5% CO_2_.

### LncRNA Microarray

Arraystar human lncRNA microarray (v4.0, KangChen Biotech, Shanghai, China) was designed to determine the expression profiling of human lncRNA and protein-coding mRNA transcripts. A total of 40,173 lncRNAs were detected in two tiered compilations, which consisted of gold standard lncRNAs for 7,506 well-annotated, functionally studied and experimentally supported full length lncRNAs, and reliable lncRNAs for 32,667 high confidence lncRNAs as the comprehensive collection. The lncRNAs were constructed using the most highly respected public transcriptome databases (Refseq, UCSC known genes, Ensembl), as well as landmark publications. The array also included an entire collection of 20,730 protein coding mRNAs that were further supported by UniProt (Universal Protein Resource) catalog. Positive probes for housekeeping genes and negative probes were printed on the array for hybridization quality control.

Total RNA was extracted from plasma samples of five ccRCC patients and five normal controls for microarray using Trizol Reagent (Invitrogen, Carlsbad, CA, USA), followed by purification using RNeasy mini kit (Qiagen, Hilden, Germany) according to the manufacturer's protocol. Sample labeling and array hybridization were performed according to the Agilent One-Color Microarray-Based Gene Expression Analysis protocol (Agilent, Santa Clara, CA, USA) with minor modifications. Briefly, five qualified RNA samples of each group were purified from total RNA after removal of rRNA (mRNA-ONLY™ Eukaryotic mRNA Isolation Kit, Epicenter). Each sample was amplified and transcribed into fluorescent cRNA along the entire length of the transcripts without 3′ bias utilizing a random priming method (Arraystar Flash RNA Labeling Kit, Arraystar). The labeled cRNA samples were then purified using RNeasy Mini Kit (Qiagen, Hilden, Germany). Concentration and specific activity of labeled cRNA samples (pmol Cy3/μg cRNA) were subsequently measured using a NanoDrop ND-1000. Labeled cRNA (1 μg) for each sample was fragmented by adding 5 μl 10 × blocking agent and 1 μl of 25 × fragmentation buffer, followed by heating at 60°C for 30 min. Finally, 25 μl 2 × GE hybridization buffer was added to dilute the labeled cRNA. Hybridization solution (50 μl) was dispensed into the gasket slide and assembled to the lncRNA expression microarray slide. The slides were incubated for 17 h at 65°C in an Agilent hybridization oven. Finally, the hybridized arrays were scanned with using a DNA Microarray Scanner (part No. G2505C, Agilent, Santa Clara, CA, USA).

Agilent Feature Extraction software (version 11.0.1.1) was used to analyze acquired array images. Quantile normalization and subsequent data processing were performed using the GeneSpring GX v12.1 software package (Agilent Technologies). Differentially expressed lncRNAs and mRNAs with statistical significance between the two groups were identified through *P*-value/false discovery rate (FDR) filtering. Differentially expressed lncRNAs and mRNAs between the two samples were identified through fold change filtering. The screening criteria for the differentially expressed genes were as follows: changes of more than 2-fold change for up-regulation or down-regulation; a *P* < 0.05; and an FDR of <0.05. The microarray data were deposited in Gene Expression Omnibus (GEO) database (accession No.:GSE150833) (https://www.ncbi.nlm.nih.gov/geo/query/acc.cgi?acc=GSE150833) in the NCBI database.

### Real-Time PCR

Total RNA was extracted from plasma, tissue and cell lines using Trizol reagent (SL2075, Coolaber). The cDNA synthesis was performed by HiScript II Q RT SuperMix for Real-Time PCR and gDNA wiper kit (R123-01, Nuoweizan Biotech, Nanjing, China) according to the manufacturer's protocol. DNA wiper was carried out at 42°C for 2 min with the reaction buffer containing 4 μl DNA wiper Mix and 500 ng template RNA. Reverse transcription was conducted in a 20 μl reaction volume containing 5 × Hiscript II qRT SuperMix II and 16 μl DNA wiper reaction.

To confirm the results obtained from microarrays, Real-Time PCR was performed to amplify the *SOCS2-AS1* in plasma and tissue of another patients and healthy controls, as well as the cell lines. Real-time PCR for *SOCS2-AS1* was carried out in a 20 μl reaction volume containing 2 × SYBR Green qPCR Mix (Q111-01/02/03, Nuoweizan Biotech, Nanjing, China), 10 μM each primer, and 1 μl cDNA template. PCR amplification conditions were as follows: 95°C for 5 min, followed by 45 cycles of 95°C for 10 s and 55°C for 30 s using LightCycler®96 (Roche, USA). All values were normalized to an endogenous β-actin control. The primer sequences were as follows: *SOCS2-AS1*: forward, 5′CTCAACGAAGAGTGTGTGGC3′; reverse, 5′ GTTCTTTGACAGGCTCCCTCC3′; β-actin: forward, 5′ TTCCAGCAGATGTGGATCAGC3′; reverse, 5 GAAGCATTTGCGGTGGAC3′. The quantification of the PCR results was performed using the 2^−ΔΔCt^ method.

### Gene Ontology (GO) and Kyoto Encyclopedia of Genes and Genomes (KEGG) Analysis

GO (www.geneontology.org) and the KEGG database (http://www.genome.ad.jp/kegg/) were used to investigate biological function and signaling pathways mediated by differentially expressed mRNAs. GO analysis was performed to facilitate the understanding of the unique biological significance of the genes in the distinctive or representative profiles. Pathway analysis of differentially expressed genes was conducted to identify the correlated pathways, based on the latest KEGG database. The significant GO terms and pathways were identified by Fisher's exact test, and FDR was utilized to correct the *P*-values. It was considered statistically significant in the presence of a *P* < 0.05.

### Bioinformatic Analysis of Differently Expressed lncRNAs and mRNAs

To identify the promising lncRNAs for the diagnosis and prognosis of ccRCC, we searched PubMed for articles involving lncRNA microarray in ccRCC tissues. To minimize the discrepancies of the batches, we selected a previous study (16) performed by the same company for the microarray assay, in which the microarray data were deposited in the ArrayExpress database (accession No. E-MTAB-1830). The seqname and gene symbol were checked separately with Venn software online to detect the differentially expressed lncRNAs between the two datasets.

Multiple Gene Expressed Tables in NetworkAnalyst (https://www.networkanalyst.ca/) were utilized to analyze the mRNA expression between the E-MTAB-1830 and our dataset. A combination of *P*-values with Fisher's method was chosen for the meta-analysis method. Then we analyzed the expression of mRNAs in patients with cancer and the normal controls, followed by survival analysis on the GEPIA online database (http://gepia.cancer-pku.cn/), which was used to analyze the effects of the commonly differentially expressed mRNAs on OS in ccRCC patients. The analysis was performed on the basis of hundreds of samples from the GTEx projects and TCGA (26). The median expression level was used as the cutoff value to divide high- and low- expression groups in the GEPIA website. To validate these differently expressed genes, the ULCAN website (http://ualcan.path.uab.edu/) was utilized (27). The analysis was conducted using the default parameters on the websites.

### Statistical Analysis

SPSS 22.0 software was used for the statistical analysis. Student's *t*-test or Wilcoxon rank sum test was used for statistical analysis of *SOCS2-AS1* in the peripheral blood samples or tissue samples from normal individuals and cancer patients, as well as the *SOCS2-AS1* expression differences in different cell lines. *P* < 0.05 was considered statistically significant.

## Results

### Characteristics of the ccRCC Patients and Healthy Controls

Five ccRCC patients and five healthy controls underwent microarray analyses, respectively. Detailed characteristics for each patient and healthy control are shown in Table 1. For the validation, no differences were noticed in the gender and age between ccRCC cases and healthy controls (all *P* > 0.05).

**Table 1:** General information of the five ccRCC patients and five healthy controls for microarray.

**Variable**	**Age/sex**	**Kidney**	**TNM stage**	**Tumor size (cm^**3**^)**	**Surgical method**	**Fuhrman grade**
**PATIENTS**						
No.1	67 y/M	Left	T2aN0M0	8.5 × 6 × 5.5	Radical nephrectomy	3
No.2	52 y/M	Right	T1bN0M0	6.5 × 6 × 5	Radical nephrectomy	2
No.3	61 y/F	Right	T1aN0M0	4 × 3.5 × 2.8	Nephron-sparing surgery	2
No.4	76 y/M	Right	T3aN0M0	4.5 × 4.5 × 3.5	Radical nephrectomy	4
No.5	66 y/F	Right	T1aN0M0	3.5 × 2.5 × 2	Radical nephrectomy	2
**HEALTHY CONTROLS**						
No.1	64 y/M	-	-	-	-	-
No.2	52 y/F	-	-	-	-	-
No.3	74 y/F	-	-	-	-	-
No.4	70 y/M	-	-	-	-	-
No.5	70 y/M	-	-	-	-	-

### Differential Expression of Plasma lncRNAs and mRNAs in ccRCC Patients and Controls

**Figure 1:**
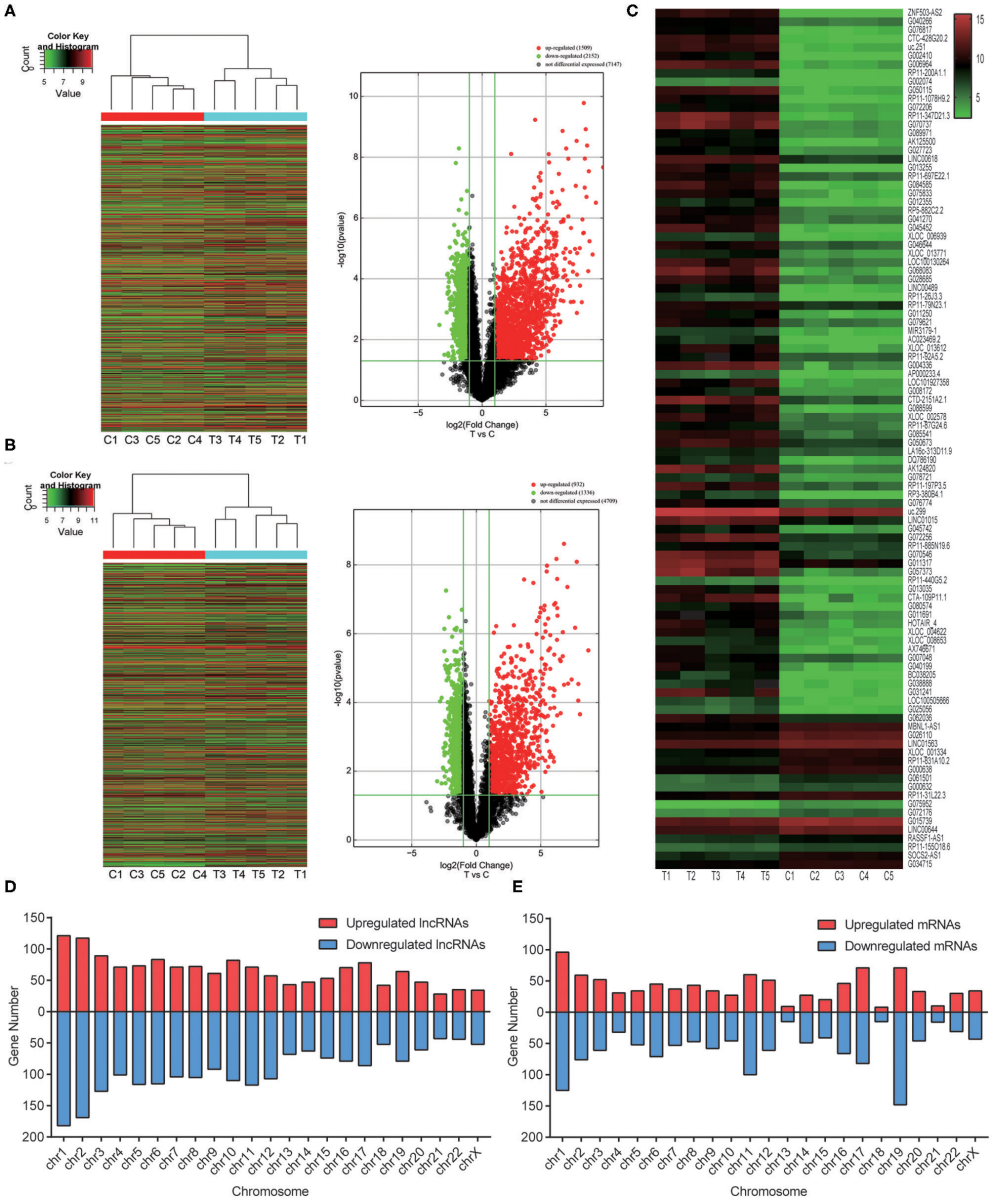
Overview of the differentially expressed lncRNAs and mRNAs based on genome-wide lncRNA microarray assay. **(A)** Heatmap results and Volcano plots of overall lncRNAs analysis between ccRCC plasma and normal plasma. **(B)** Heatmap results and Volcano plots of overall protein-coding mRNAs analysis between ccRCC plasma and normal plasma samples. **(C)** Heatmap results of significantly changed top 100 lncRNAs. **(D)** The distribution of overall lncRNAs on human chromosomes. **(E)** The distribution of overall protein-coding mRNAs on human chromosomes.

Genome-wide plasma lncRNA microarray analysis was conducted to detect differential lncRNA expression between ccRCC cases and healthy controls. After data normalization, a total of 3,664 differentially expressed lncRNAs were revealed by heatmap and volcano-plot, including 1,511 up-regulated and 2,153 down-regulated lncRNAs (fold change ≥ 2 and *P* < 0.05, Figure 1A, Supplementary Table 1), respectively. Compared with healthy controls, differential expression was noticed in 2,268 mRNAs, including 932 up-regulated mRNAs and 1,336 down-regulated mRNAs, respectively (fold change ≥ 2 and *P* < 0.05, Figure 1B, Supplementary Table 2). Among them, significant up-regulation (>10-fold) was noticed in 514 lncRNAs and 259 mRNAs in ccRCC plasma. Meanwhile, significant down-regulation (> 5-fold) was noticed in 31 lncRNAs and 22 mRNAs in ccRCC plasma. We preferentially selected the top 20 up-regulated and top 20 down-regulated lncRNAs and mRNAs (Tables 2, 3). The top 100 deregulated lncRNAs were presented in Figure 1C.

**Table 2:** Top 20 differentially expressed lncRNAs determined by microarray.

**Seqname**	**GeneSymbol**	***P*-value**	**FDR**	**Log_**2**_ fold change**
**UPREGULATED**				
ENST00000428999	RP11-347D21.3	0.0000000214	0.000015454	9.4819463
T291258	G068083	0.0000003147	0.000092339	8.91107676
T292627	G068474	0.0000158625	0.001119352	8.65774498
T133054	G031241	0.0000058091	0.00064719	8.4121885
T302937	G070737	0.0000000290	0.000019611	8.36001398
T222083	G051327	0.0000560055	0.001913021	8.31355126
T030889	G006964	0.0000000041	0.000006989	8.22694142
T197170	G045452	0.0000002022	0.00007284	8.17947228
ENST00000514544	CTC-428G20.2	0.0000000012	0.000003708	8.11873496
T216491	G050115	0.0000000111	0.000010064	8.04823386
T358504	G084585	0.0000001003	0.00004712	8.03782684
T249502	G057373	0.0000030645	0.000415286	7.96898692
NR_024421	ZNF503-AS2	0.0000000002	0.000001788	7.96820554
ENST00000450247	CTA-109P11.1	0.0000031465	0.000419845	7.92116788
T019592	G004336	0.0000011153	0.00023636	7.86037754
ENST00000566945	CTD-2199O4.3	0.0005875201	0.00492951	7.8087944
T034301	G007801	0.0001678137	0.002963612	7.6693759
T050410	G011795	0.0000098004	0.000851873	7.65105836
T150043	G034777	0.0000142887	0.001071793	7.4421959
T077631	G017987	0.0000728074	0.002121031	7.43471032
**DOWNREGULATED**				
NR_104628	LOC101929681	0.0032868811	0.013022218	3.340665419
T157700	G036324	0.0314183916	0.065402538	2.949606851
uc.336-	uc.336	0.0012743187	0.007337686	2.76525229
T193188	G044493	0.0000733166	0.002130123	2.735163474
ENST00000570186	RP11-203B7.2	0.0003123646	0.003898426	2.705130896
T186396	G042787	0.0002771637	0.003716463	2.687304716
T004817	G000950	0.0000677791	0.002057742	2.686282678
T107545	G025378	0.0281952413	0.060188459	2.652377202
T224378	G051828	0.0001860083	0.00315016	2.652073188
TCONS_00012383	XLOC_005950	0.0344873162	0.070090055	2.635172228
T381125	G090339	0.0001270249	0.002610048	2.593713288
T234995	G054048	0.0002567889	0.003585755	2.586390927
TCONS_00023307	XLOC_011177	0.0000929609	0.002332051	2.57338238
ENST00000568585	RP11-440L14.3	0.0004564715	0.004449114	2.566904903
NR_034108	TRAF3IP2-AS1	0.0004352604	0.004376088	2.545508239
ENST00000470758	RP5-1002M8.4	0.0000092200	0.000837395	2.528888827
T067091	G015577	0.0010290270	0.006584798	2.490012506
T013102	G002824	0.0007792867	0.005675049	2.485516709
NR_110847	LOC101928674	0.0004754173	0.004530038	2.463422961
ENST00000425358	HOTAIRM1	0.0006508989	0.005175843	2.45984459

**Table 3:** Top 20 differentially expressed mRNAs determined by microarray.

**Seqname**	**GeneSymbol**	***P*-value**	**FDR**	**Log_**2**_ fold change**
**UPREGULATED**				
NM_001190708	MTRNR2L10	0.0000030720	0.000420261	8.68257776
NM_015888	HOOK1	0.0002209570	0.003038212	8.03730918
NM_005157	ABL1	0.0000291098	0.001279824	7.90943988
NM_017670	OTUB1	0.0000887645	0.002157875	7.90113294
NM_001013732	PTCHD4	0.0000000082	0.000018452	7.78691172
NM_001098529	TXNDC2	0.0000006739	0.000163298	7.6578123
uc010nsc.1	GPR112	0.0000215860	0.001091346	7.38992936
NM_024672	THAP9	0.0000285122	0.001279824	7.11422588
NM_024824	ZC3H14	0.0000000440	0.000034118	7.061392279
NM_001005480	OR2A2	0.0000308317	0.001279824	6.7989647
NM_005987	SPRR1A	0.0000000024	0.000017085	6.79420714
NM_001001933	LHX8	0.0000009086	0.000181129	6.7080964
NM_001114086	CLIC5	0.0000045417	0.000518031	6.70282074
NM_153217	TMEM174	0.0000063097	0.000586269	6.542464901
ENST00000434783	FAM230A	0.0002060010	0.002936016	6.539820479
NM_002170	IFNA8	0.0000056067	0.000566155	6.48139208
NM_017669	ERCC6L	0.0000003014	0.000110681	6.29564714
NM_002235	KCNA6	0.0000001811	0.00008423	6.27521024
NM_001042705	IQCJ	0.0000000257	0.000026544	6.270433501
NM_024628	SLC12A8	0.0002387190	0.003136867	6.270391981
**DOWNREGULATED**				
ENST00000600684	ZSCAN5D	0.0196384170	0.045550943	3.033314789
NM_198990	NAPEPLD	0.0000932634	0.002204989	2.803487111
ENST00000589616	LPHN1	0.0225998089	0.050749555	2.689420252
ENST00000427721	RP11-295K3.1	0.0000734431	0.002017372	2.684541592
NM_052907	TMEM132B	0.0078601564	0.023466115	2.578530185
NM_153611	CYB561A3	0.0083900956	0.024554403	2.553794869
NM_015353	KCTD2	0.0021048383	0.010045066	2.551246771
NM_000816	GABRG2	0.0027924807	0.011872474	2.474187149
NM_016571	LGSN	0.0000007260	0.000168849	2.47368014
NM_032310	C9orf89	0.0000032474	0.000425491	2.471114608
ENST00000450895	AP000322.53	0.0034442230	0.013599515	2.462454751
NM_021922	FANCE	0.0142285452	0.035748131	2.44960239
NM_005380	NBL1	0.0000084567	0.000670478	2.438026807
NM_012333	MYCBP	0.0003447929	0.003699849	2.426022093
NM_021911	GABRB2	0.0015156797	0.008223093	2.418129702
NM_014266	HCST	0.0018845135	0.009371526	2.404002219
NM_001142964	C22orf46	0.0223272839	0.050250793	2.38353203
NM_001077351	RBM23	0.0000105011	0.000772095	2.365747904
NM_017457	CYTH2	0.0028788866	0.012091734	2.362240526
NM_016932	SIX2	0.0016260163	0.008549145	2.354043555

To reveal potential roles of lncRNAs in ccRCC, we analyzed the distribution of lncRNAs and mRNAs. In addition, the relationship between differentially expressed lncRNAs and their adjacent protein-coding genes was investigated. The transcripts located in chromosome Y were excluded to eliminate the effects of gender. These lncRNAs and mRNAs were widely distributed in all chromosomes (Figures 1D,E). The well-annotated 1,192 lncRNAs that were differentially expressed were classified into 6 categories: natural antisense (15.86%), intronic antisense (13.84%), intron sense-overlapping (3.86%), bidirectional (3.52%), and exon sense-overlapping (1.68%). Intergenic lncRNAs constituted the largest number in all differentially expressed lncRNAs (61.24%). There were overlaps between natural antisense and intronic antisense lncRNAs.

### GO and KEGG Pathway Analysis of Deregulated mRNAs

**Figure 2:**
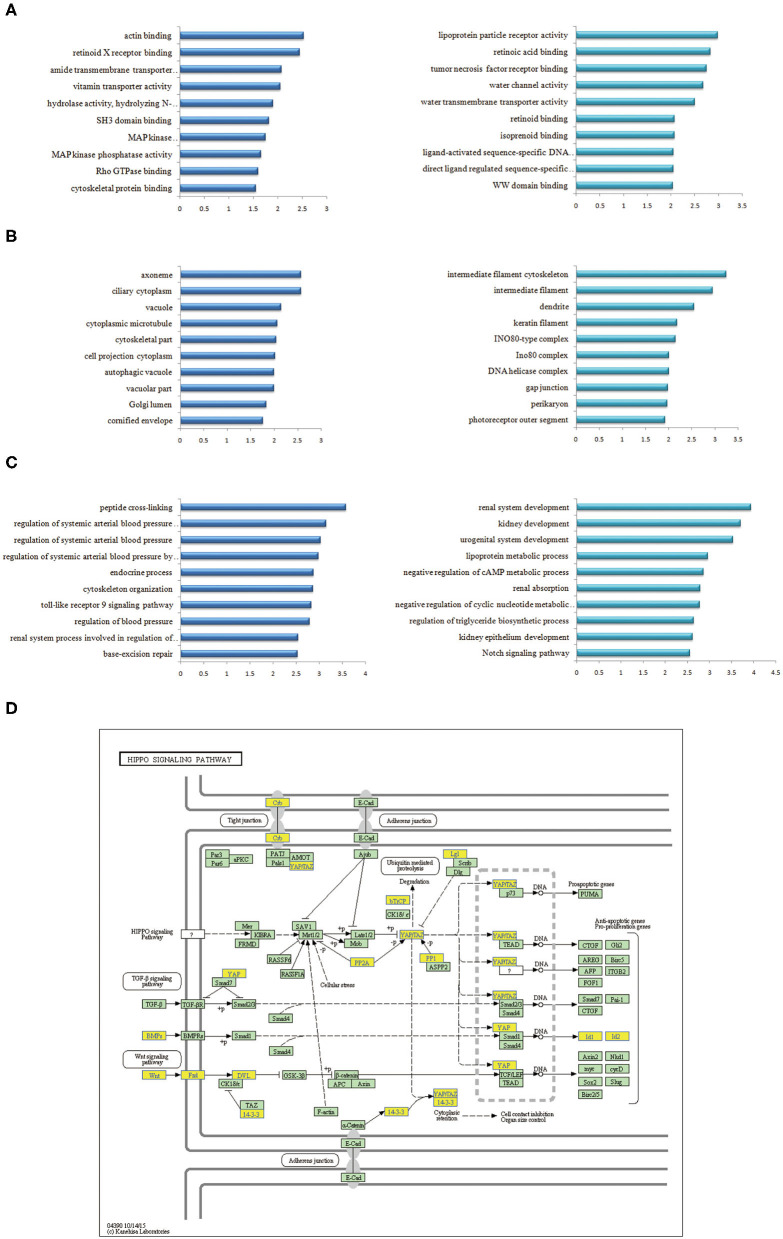
GO and KEGG pathway analyses of differentially expressed mRNAs in plasma of ccRCC. **(A)** Go annotations of up- and down- regulated mRNAs with top 10 enrichment scores of molecular function. **(B)** Go annotations of up-regulated and down-regulated mRNAs with top 10 enrichment scores of cellular component. **(C)** Go annotations of up-regulated and down-regulated mRNAs with top 10 enrichment scores of biological process. **(D)** KEGG pathway annotations of Hippo pathway. Yellow marked nodes indicated down-regulated genes, while green nodes indicated genes of no significance.

GO analysis indicated that the most significantly enriched molecular function of up-regulated plasma mRNAs of ccRCC patients was associated with actin binding, retinoid X receptor binding, and amide transmembrane transporter activity (Figure 2A left). The down-regulated genes were mainly involved in lipoprotein particle receptor activity, retinoic acid binding, and tumor necrosis factor receptor binding (Figure 2A right). For cellular components, the up-regulated mRNAs were significantly enriched in ciliary cytoplasm, axoneme, and vacuole (Figure 2B left), while the down-regulated mRNAs were significantly enriched in intermediate filament cytoskeleton, intermediate filament, and dendrite (Figure 2B right). The most significantly enriched biological process of the up-regulated mRNAs were peptide cross-linking, regulation of systemic arterial blood pressure mediated by a chemical signal, and regulation of systemic arterial blood pressure (Figure 2C left). Nevertheless, the down-regulated mRNAs were enriched in renal system development, renal development and urogenital system development (Figure 2C right, Table 4).

**Table 4:** The most significantly enriched biological process of downregulated genes in plasma of ccRCC patients and health controls.

**Term**	**Count**	***P*-value**	**Genes**
Renal system development	33	0.000116503	CTSH//FGF10//GLI3//ID2//ARG2//SMAD6//RARA//PBX1//CTNNBIP1//BMP2//WNT1//WNT6//SIX2//TMED10//TENC1//SLC12A1//HYAL2//PYGO2//STAT1//HEYL//MPV17//CALB1//TP63//ANGPT1//KIF26B//NOTCH3//ACTA2//KLHL3//TBX18//AQP1//LAMB2//YAP1//EDNRA
Kidney development	31	0.000200874	CTSH//FGF10//GLI3//ID2//ARG2//SMAD6//RARA//PBX1//CTNNBIP1//BMP2//WNT1//WNT6//SIX2//STAT1//HEYL//MPV17//CALB1//ANGPT1//KIF26B//NOTCH3//ACTA2//KLHL3//AQP1//LAMB2//YAP1//EDNRA//TMED10//TENC1//SLC12A1//HYAL2//PYGO2
Urogenital system development	35	0.000299161	CTSH//FGF10//GLI3//ID2//ARG2//SMAD6//RARA//PBX1//CTNNBIP1//BMP2//WNT1//WNT6//SIX2//TMED10//TENC1//SLC12A1//HYAL2//PYGO2//STAT1//HEYL//MPV17//CALB1//TP63//HOXB13//ANGPT1//KIF26B//NOTCH3//ACTA2//KLHL3//TBX18//AQP1//LAMB2//YAP1//EDNRA//EPHB2
Lipoprotein metabolic process	17	0.001083699	PPT1//ALOX12B//PLAUR//PIGV//PIGQ//PIGZ//PIGY//CLIP3//ZDHHC15//ZDHHC22//ZDHHC1//LDLR//HHATL//HSPG2//OLR1//APOL1//SCARB1
Negative regulation of cAMP metabolic process	8	0.001376599	GRM7//PALM//CCR2//EDNRA//APLP1//NPY2R//AKAP6//SSTR4
Renal absorption	5	0.001632582	KLHL3//AQP1//AQP3//AQP4//HYAL2
Negative regulation of cyclic nucleotide metabolic process	8	0.001662108	GRM7//PALM//CCR2//SSTR4//EDNRA//APLP1//NPY2R//AKAP6
Regulation of triglyceride biosynthetic process	5	0.002254161	NR1H3//LDLR//PLIN5//SCARB1//FBXW7
Kidney epithelium development	18	0.002436187	ARG2//SMAD6//RARA//GLI3//PBX1//CTNNBIP1//BMP2//WNT1//WNT6//SIX2//STAT1//CALB1//HEYL//KIF26B//KLHL3//AQP1//LAMB2//YAP1
Notch signaling pathway	20	0.002758315	NOTCH4//FBXW7//NUMB//DLK1//NEURL1//FGF10//WNT1//TP63//ITGB1BP1//HDAC5//E2F3//ETV2//HEYL//NOTCH3//NEURL1B//TMEM100//TNRC6C//BMP2//TLE2//TLE3

KEGG pathway analysis indicated that the up-regulated mRNAs in the plasma of ccRCC patients were mostly enriched in base excision repair, salmonella infection, pentose and glucuronate interconversions. The down-regulated mRNAs were significantly associated with the melanogenesis, Hippo signaling pathway (Figure 2D) and vascular smooth muscle contraction (*P* < 0.05) after multiple testing correction.

### Validation of Microarray Results by Real-Time PCR

**Figure 3:**
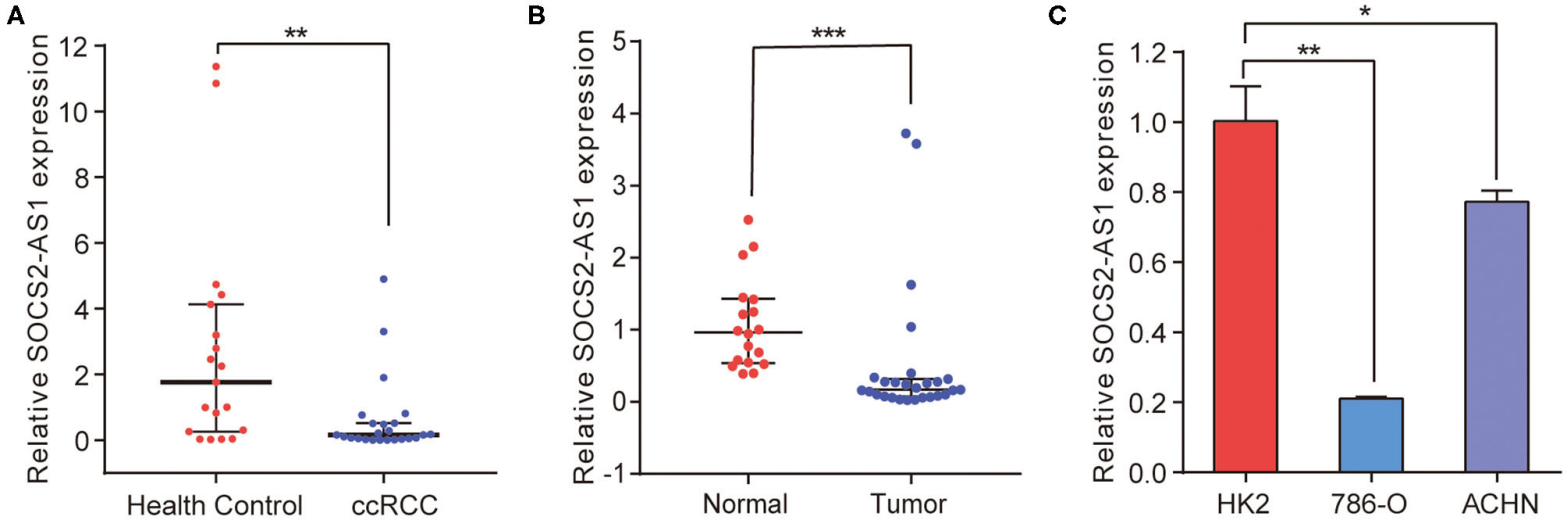
Real-Time PCR for SOCS2-AS1 in ccRCC plasma, tissue and cell lines. **(A)** SOCS2-AS1 expression in the plasma from ccRCC patients and health controls. **(B)** SOCS2-AS1 in the ccRCC neoplastic tissues and normal controls. **(C)** Expression of SOCS2-AS1 in HK2 and ccRCC cell lines. *P < 0.05; **P < 0.01; ***P < 0.001.

To validate the microarray data, NR_038263 (SOCS2-AS1) was selected for confirmation of microarray results using Real-Time PCR in the validation cohort, which consisted of 24 ccRCC patients (Table 5) and 19 healthy controls. The NR_038263 (SOCS2-AS1) was then selected based on the fold changes in expression (fold changes > 2, *P* < 0.05), the length of lncRNAs (length <1,000 bp), and whether the lncRNAs presented definite sequences. Real-Time PCR results confirmed that the expression level of plasma NR_038263 (SOCS2-AS1) showed a significant decrease in ccRCC patients (*P* = 0.004, Figure 3A). The results were consistent with those obtained by microarray analysis.

**Table 5:** Clinical characteristics of ccRCC patients for validation in plasma and tissue.

	**Plasma cases (*n* = 24)**	**Tissue cases (*n* = 27)**
**SEX**		
Male	14/24 (58.33%)	16/27 (59.26%)
Female	10 /24 (41.67%)	11/27 (40.74%)
Age (years)	31–76	32–71
Mean age (years)	60 ± 11.74	58 ± 9.73
≤60 years	13/24 (51.17%)	17/27 (62.96%)
>60 years	11/24 (45.83%)	10/27 (37.04%)
**SIZE**		
≤4 cm	10/24 (41.67%)	7/27 (25.93%)
>4 cm	14/24 (58.33%)	20/27 (74.07%)
**TNM STAGE**		
pT1	19/24 (79.17%)	17/27 (62.96%)
pT2	2/24 (8.33%)	6/27 (22.22%)
pT3	3/24 (12.50%)	3/27 (11.11%)
pT4	0/24 (0%)	1/27 (3.70%)
**FUHRMAN GRADE**		
G1	3/24 (12.50%)	3/27 (11.11%)
G2	16/24 (66.67%)	13/27 (48.15%)
G3	4/24 (16.67%)	9/27 (33.33%)
G4	1/24 (4.17%)	2/27 (7.41%)
Lymph node metastasis	0/24 (0%)	0/27 (0%)
**VASCULAR INVASION**		
Yes	1/24 (4.17%)	0/27 (0%)
No	23/24 (95.83%)	27/27 (100%)
**DISTANT METASTASIS**		
Yes	0/24 (0%)	1/27 (3.70%)
No	24/24 (100%)	26/27 (96.30%)

We further analyzed the expression of NR_038263 (SOCS2-AS1) in 27 ccRCC tissues and 18 paired normal kidney tissues (Table 5). Expression of NR_038263 (SOCS2-AS1) was significantly lower in ccRCC tissues compared to that of the normal tissues (*P* < 0.0001, Figure 3B). The expression of NR_038263 (SOCS2-AS1) was significantly lower in 786-O (*P* = 0.005) and ACHN (*P*= 0.045, Figure 3C) compared to that of the HK2 cells.

### Bioinformatics Analysis of Deregulated lncRNAs and mRNAs

E-MTAB-1830 in ArrayExpressed databases was selected upon inspection of the microarray data in PubMed as both microarrays were done by the same company with the same series of microarray. The E-MTAB-1830 dataset was conducted with Arraystar Human LncRNA Microarray v2.0 which was performed by KangChen Biotech (Shanghai, China). The Venn diagram software was utilized to identify the differentially expressed genes (DEGs) that were the same in the two datasets by seqname and gene symbol, respectively. A total of 18 common DEGs were detected (Table 6). NR_027011, NR_027471, uc.263-, and ENST00000442072 were highly expressed in the neoplasm and the plasma of ccRCC patients. NR_024256, ENST00000478814, and ENST00000436529 were down-regulated in the neoplasm and the plasma of ccRCC patients. ENST00000456082, ENST00000417084, ENST00000450618, NR_024046, and ENST00000457632 were up-regulated in the E-MTAB-1830 and down-regulated in our study. The expression of H19, ENST00000413094, ENST00000439438, ENST00000443034, ENST00000448001, and NR_024054 was down-regulated in the E-MTAB-1830 and was up-regulated in our study.

**Table 6:** Eighteen different expressed lncRNAs detected by Venn software online between E-MTAB-1830 and our dataset.

**Seqname**	**Gene symbol**	**E-MTAB-1830**			**This study**			**RNA length**	**Chrom**	**Relationship**
		***P* value**	**Fold change**	**Regulation**		***P* value**	**Fold Change**	**Regulation**			
NR_027471	LOC440173	0.030198595	2.429401	Up	0.037620397	12.762367	Up	2018	chr9	Intergenic
uc.263-	uc.263	0.02442751	1.7595506	Up	0.001260717	37.5296799	Up	207	chr9	Exon sense- overlapping
ENST00000442072	RP11-440G5.2	0.026290732	1.7149061	Up	0.000002844	6.4797364	Up	474	chr9	Intergenic
NR_027011	YBX3P1	0.04660399	1.6085646	Up	0.032184332	2.8030689	Up	1758	chr16	Intergenic
ENST00000417084	RP11-6J21.2	0.039214045	1.5371555	Up	0.000749542	3.1189851	Down	917	chr1	Intergenic
NR_024046	NRADDP	0.03138734	1.6566974	Up	0.026993902	2.18563	Down	1012	chr3	Intergenic
ENST00000450618	RP3-340B19.3	0.033042107	1.5136869	Up	0.000347281	2.2356763	Down	595	chr6	Intergenic
ENST00000456082	RP11-478H13.1	0.012399554	1.8661174	Up	0.00182004	2.8583647	Down	517	chr10	Intergenic
ENST00000457632	RP11-799O21.2	0.038610306	2.700243	Up	0.001679587	2.3802945	Down	299	chr10	Intergenic
ENST00000443034	RP5-1092A11.5	0.032418218	3.870721	Down	0.006580169	3.9530714	Up	754	chr1	Intronic antisense
ENST00000448001	RP3-380B4.1	0.021664698	4.047757	Down	0.000002382	26.3881817	Up	458	chr1	Intergenic
NR_024054	SMA4	0.011396141	1.5648494	Down	0.002857332	8.9064164	Up	1010	chr5	Intergenic
ENST00000413094	RP11-3P22.1	0.000949942	2.71848	Down	0.001267507	12.5110581	Up	439	chr7	Intergenic
ENST00000439438	RP11-305L7.6	0.01463832	1.5166456	Down	0.000044447	3.1932374	Up	454	chr9	Intergenic
NR_002196	H19	0.004748824	1.7570378	Down	0.000077220	14.0456128	Up	2356	chr11	Intergenic
ENST00000478814	RP11-439C8.1	0.047738522	1.5696678	Down	0.000326084	3.1831687	Down	351	chr3	Intergenic
NR_024256	GATA3-AS1	0.03323272	12.378197	Down	0.006413275	2.8931782	Down	2214	chr10	Intergenic
ENST00000436529	BACH1-IT2	0.044178534	2.297524	Down	0.003093362	4.720694	Down	388	chr21	Intergenic

**Figure 4:**
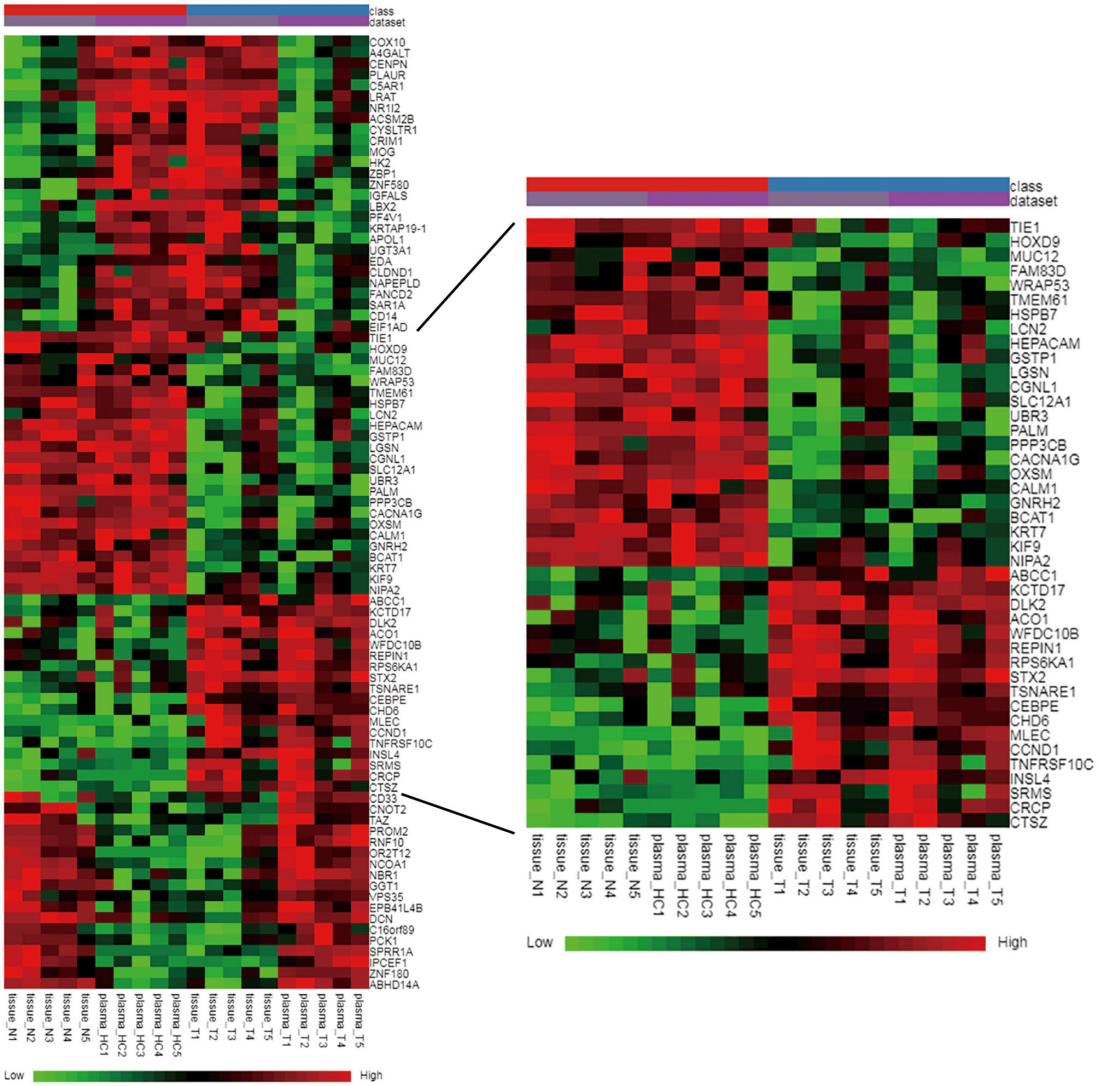
Hierarchical clustering heat map of the common differently expressed mRNAs between E-MTAB-1830 and our datasets. Green color: low expression; black color: moderate expression; red color: high expression.

**Figure 5:**
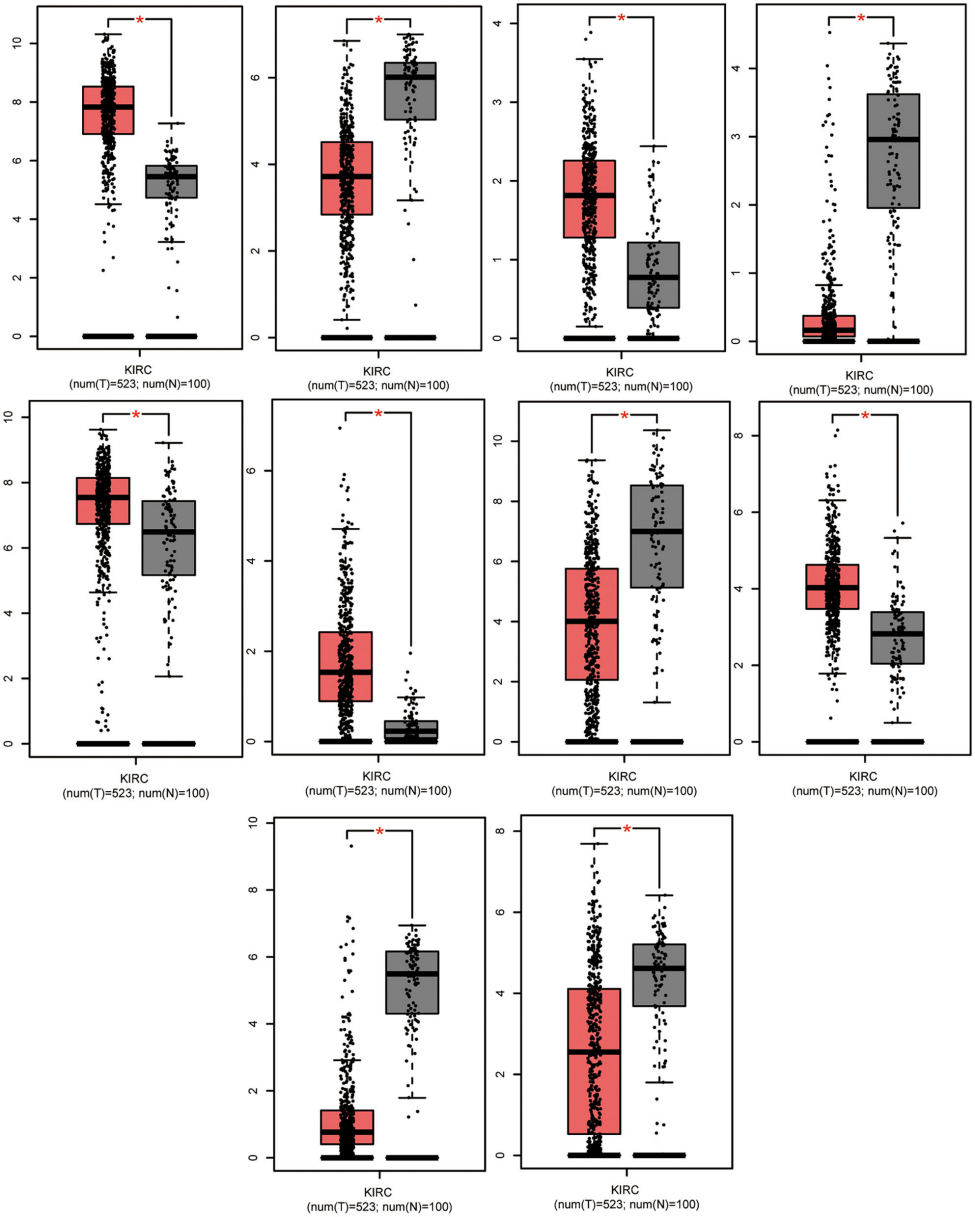
Significant expression of *EPB41L4B, CCND1, GGT1, CGNL1, CYSLTR1, PLAUR, UGT3A1, PROM2, MUC12*, and *PCK1* in ccRCC tissues compared to normal. Data were analyzed by GEPIA. *P < 0.05.

**Figure 6:**
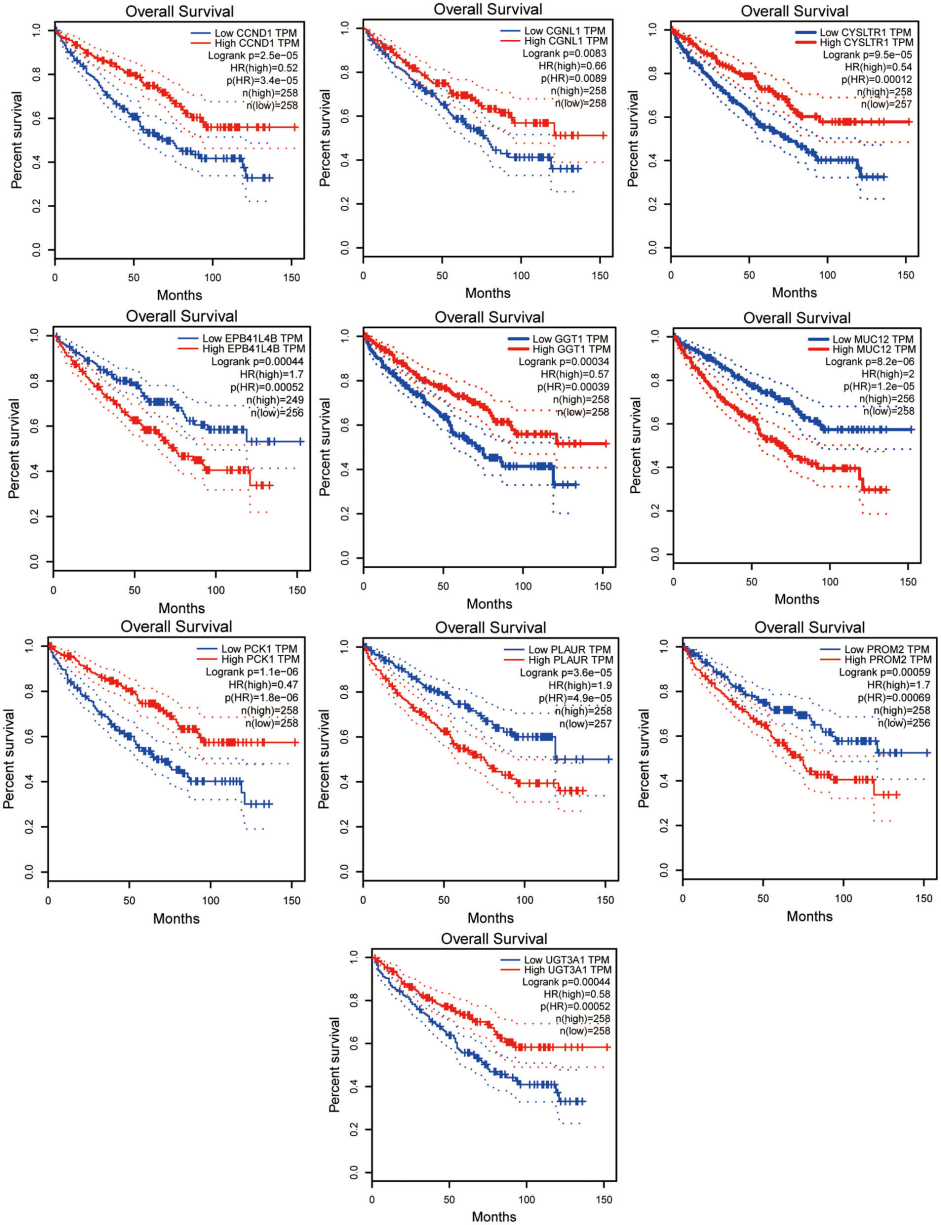
Survival curve of *EPB41L4B, CCND1, GGT1, CGNL1, CYSLTR1, PLAUR, UGT3A1, PROM2, MUC12*, and *PCK1*. All of these genes were correlated with OS of ccRCC patients. Data were analyzed by GEPIA.

In total, 87 commonly differentially expressed mRNAs were obtained through analysis in the Multiple Gene Expressed Tables of NetworkAnalyst (Figure 4 left). Eighteen mRNAs were up-regulated in the neoplastic tissues and plasma of ccRCC patients, while 24 mRNAs were down-regulated in the neoplastic tissues and plasma of ccRCC patients (Figure 4 right). The expression patterns of the rest of the mRNAs were not consistent between the neoplasm tissues and the plasma. GEPIA and ULCAN were utilized to analyze the 87 mRNAs that were differentially expressed, as well as their correlation with the prognosis. Twenty-three genes (23/87) showed differential expression in GEPIA and ULCAN websites (Figure 5, Supplementary Figure 1). For the OS rate analysis of the 23 genes, GEPIA and ULCAN indicated that 10 genes (*EPB41L4B, CCND1, GGT1, CGNL1, CYSLTR1, PLAUR, UGT3A1, PROM2, MUC12*, and *PCK1*) may play a key role in the OS rate in patients with ccRCC (*P* < 0.05, Figure 6, Supplementary Figure 2). Among these genes, *CCND1, GGT1, CGNL1, CYSLTR1, UGT3A1*, and *PCK1* were positively correlated with the OS rate in ccRCC patients. Additionally, *EPB41L4B, MUC12, PLAUR*, and *PROM2* were negatively correlated with the OS rate in ccRCC patients.

## Discussion

RCC accounts for more than 90% of all renal malignancies and 30% of patients are at the advanced stage upon diagnosis. Additionally, 20% of patients showed tumor progression and relapse after radical surgery. The median survival of patients with metastatic RCC was only 6–12 months, and only a small number of patients (9%) showed a survival duration of 5 years. This was highly related to the resistance to chemotherapy and radiotherapy (28). Therefore, earlier diagnosis and treatment is crucial to improve life quality and prolong survival. To date, few tumor biomarkers have been identified for the screening of renal cancer. Irregular regulation of lncRNAs has been shown to be associated with the pathogenesis of several human cancers. These lncRNAs were specifically expressed in cells localized in sub-cellular compartments (16). Altered lncRNA expression patterns were reported to be associated with tumorigenesis and malignancy transformation in ccRCC.

In the past decades, several studies had focused on the lncRNA expression profiles in ccRCC tissues (14–23). For example, Brito et al. first reported a subset of down-regulated intronic non-coding RNAs in six ccRCC patients (29). In addition, Fachel et al. uncovered a signature of 29 intronic lncRNAs that were differentially expressed between RCC and non-tumorous samples based on the combination of microarray and large-scale public data. Meanwhile, 26 intronic lncRNAs were significantly correlated with the 5-years survival rate (15). These two studies focused on the intronic lncRNA, while the other studies involved the microarray containing all lncRNA subgroups. Furthermore, the samples used in these studies were classified into different TNM stages. Li et al. identified a novel lncRNA termed metastatic renal cell carcinoma-associated transcript 1 (MRCCAT1) by microarray analysis (19). Based on the lncRNA Promoter Microarray and combined analysis of the lncRNAs expression profiles, Zhou et al. identified a series of down-regulated lncRNAs with hypermethylated promoter regions (24). To our best knowledge, few systemic studies have focused on the lncRNAs in plasma of ccRCC patients. To identify the differentially expressed lncRNAs, microarray analysis was used to determine the lncRNAs in the plasma of ccRCC patients and healthy controls, which indicated up-regulation of 1,511 lncRNAs and down-regulation of 2,153 lncRNAs (fold change ≥ 2 and *P* < 0.05).

In this study, many novel lnRNA transcripts were up-regulated in the plasma of ccRCC patients compared with healthy controls. In the previous studies, the expression of lncRNA H19 was significantly higher in ccRCC compared to the adjacent normal renal tissues (30, 31). Similarly, H19 was up-regulated in our dataset. In addition, the ccRCC patients with higher H19 expression were at a more advanced clinical stage with poorer prognosis than those with a low H19 level. Kaplan-Meier analysis revealed that patients with higher H19 expression had a poorer OS. This implied that H19 expression could be an independent prognostic marker for ccRCC (30). Moreover, H19 regulated E2F1 expression by competitively sponging endogenous miR-29a-3p in ccRCC patients (31). The expression of nuclear enriched abundant transcript 1 (NEAT1) was up-regulated in ccRCC tissues. As previously described, NETA1 up-regulation was positively correlated with tumor size, higher Fuhrman grade, lymph node metastasis, as well as a poor 5-years survival rate (32). Besides, it was up-regulated in the plasma of ccRCC patients compared with the plasma of health control. NEAT1 knock-down involved in the suppression of cell invasion and migration, as well as inhibition of the mRNA and protein expression of epithelial mesenchymal transition (EMT) related markers in ccRCC cell lines (32). Thus, NEAT1 may serve as an important mediator in regulating ccRCC progression and prognosis prediction in ccRCC patients (32). The lncRNA actin filament-associated protein 1-antisense RNA 1 (AFAP1-AS1), which was up-regulated in a variety of tumors, was associated with poor prognosis of several cancers, including lung cancer, breast cancer, and ovarian cancer (33). Its expression was significantly up-regulated in ccRCC tissues, which was up-regulated in plasma of ccRCC patients in this study. Meanwhile, patients with high-level expression of AFAP1-AS1 had a shorter OS (33). AFAP1-AS1 silencing inhibited cell proliferation, EMT and metastasis through the PTEN-dependent signaling pathway (33). On this basis, it was speculated that AFAP1-AS1 might be a novel potential biomarker for the treatment of ccRCC. DNM1P35 was found to be significantly correlated with the OS of ccRCC patients (34) and DNM1P35 was up-regulated in plasma of our ccRCC patients. These results confirmed the reliability of the microarray, which may serve as promising biomarkers for the screening and treatment of ccRCC.

In ccRCC patients, significant down-regulation was noticed in several lnRNA transcripts compared with health controls in our study. This was similar to the previous publications in which these lncRNA transcripts were down-regulated in the ccRCC neoplastic tissues according to the published data. ADAM metallopeptidase with thrombospondin type 1 motif nine antisense RNA 2 (ADAMTS9-AS2) was down-regulated in ccRCC plasma in our study. This lncRNA was down-regulated in ccRCC tissue samples and cell lines. Low ADAMTS9-AS2 level was correlated with a poorer OS in ccRCC patients (35). Meanwhile, Song et al. indicated that ADAMTS9-AS2 inhibited the ccRCC progression and impaired the chemoresistance of ccRCC via miR-27a-3p-mediated regulation of FOXO1, which may serve as a prognostic biomarker and therapeutic target for ccRCC (35). HOXA Transcript Antisense RNA Myeloid-Specific 1 (HOTAIRM1) was down-regulated in plasma of our ccRCC patients. HOTAIRM1 transcripts were induced during renal lineage differentiation of embryonic stem cells and were essential for expression of specific renal genes. Hamilton et al. showed that the major HOTAIRM1 transcript in differentiated cells was the spliced cytoplasmic HM1-3 isoform, and HM1-3 was down-regulated in >90% of ccRCC patients (36). The pervasive down-regulation of the specific HOTAIRM1 cytoplasmic isoform HM1-3 in ccRCC may play possible roles in kidney differentiation and suppression of HIF1-dependent angiogenic pathways (36). LncRNA taurine up-regulated gene 1 (lncRNA TUG1) (37, 38) and small nucleolar RNA host gene 14 (SNHG14) (39), that were down-regulated in our dataset, had been reported to be down-regulated in ccRCC tissues, which also played important roles in the pathogenesis of RCC.

Biomarkers derived from the circulating system are non-invasive for the diagnosis of tumor. The lncRNAs may be used as non-invasive biomarkers for the screening of ccRCC. However, there are only few lncRNAs in plasma of ccRCC. Wu et al. determined the expression of 91 cancer-associated lncRNA molecules in serum from ccRCC patients, and then established 5 lncRNA signatures (i.e., lncRNA-LET, PVT1, PANDAR, PTENP1 and lnc00963) serving as potential markers for discriminating ccRCC patients from healthy controls. The 5 lncRNA panel contributed to the early stage prediction of ccRCC (25).

To validate the microarray, we selected NR_038263 (suppressor of cytokine signaling 2-antisense transcript 1, SOCS2-AS1) located on chromosome 12 using Real-Time PCR in the validation cohort consists of plasma, tissues and cell lines. SOCS2-AS1 was significantly lower in plasma, tissues, as well as 786-O and ACHN cells. Meanwhile, Misawa et al. (40) investigated the expression of SOCS2-AS1 in LNCaP and VCaP cells as an androgen-regulated lncRNA. The expression of SOCS2-AS1 was higher in long-term androgen-deprived (LATD) cells, which were castration-resistant prostate cancer cells derived from LNCaP and VCaP cells. Moreover, *SOCS2-AS1* promoted cell growth and migration and inhibited the expression of several genes associated with the apoptosis pathway. This implied that androgen-induced *SOCS2-AS1* played an important role in the pathogenesis of castration-resistant prostate cancer by inhibiting cellular apoptosis (40).

To identify the prognostic biomarkers of ccRCC, bioinformatical methods were utilized based on the published (E-MTAB-1830 dataset) and our datasets. There were 18 lncRNAs and 87 mRNAs that were differentially expressed after Venn and NetworkAnalyst analysis (*P* < 0.05). These might contribute to the diagnostic amplification in ccRCC. Besides, up-regulation of four lncRNAs and 18 mRNAs, together with down-regulation of three lncRNAs and 24 mRNAs, were observed in plasma and neoplastic samples in ccRCC patients. In this study, the expression of ENST00000456082, ENST00000417084, ENST00000450618, NR_024046, and ENST00000457632 was up-regulated and the expression of E-MTAB-1830 was down-regulated. In addition, the expression of H19, ENST00000413094, ENST00000439438, ENST00000443034, ENST00000448001, and NR_024054 was down-regulated, while the E-MTAB-1830 was up-regulated. Wu et al. also noticed inconsistencies in *MALAT1, GAS5* and *KCNQ1OT1* gene expressions between tissue samples and serum samples. There were significant differences in tissues, whereas, the serum samples showed a detection rate of <50% or even no differences (25). We speculated that the potential mechanisms were as follows: (a) the alteration of expression may contribute to the escape of cancer cells from the immune responses; (b) there might be degradation of some lncRNAs and mRNAs in the plasma; (c) the experimental bias may be partially responsible for this. Furthermore, based on the Kaplan-Meier plotter analysis in GEPIA and ULCAN websites, *EPB41L4B, CCND1, GGT1, CGNL1, CYSLTR1, PLAUR, UGT3A1, PROM2, MUC12*, and *PCK1* may be associated with the OS rates in ccRCC patients. *Ehm2*, also known as erythrocyte membrane protein band 4.1-like protein 4B (*EPB41L4B*), is a member of the NF2/ERM/4.1 superfamily. In metastatic cancer cells, there was over-expression of *Ehm2* (41). Plasminogen activator urokinase receptor (*PLAUR*) played an important role in cell proliferation, migration and apoptosis. The exosomal *PLAUR* mRNA in the plasma of gefitinib-resistant NSCLC patients showed significant increase compared to that of gefitinib-sensitive NSCLC patients. *PLAUR* could be a novel therapeutic target for gefitinib-resistant NSCLC patients (42). *MUC12* was reported as a novel membrane-associated mucin gene located on chromosome 7q22 (43). *MUC12* expression was a novel independent prognostic variable in patients with stages II or III colorectal cancer (44). However, their roles in the pathogenesis of ccRCC are still not well defined.

There are some limitations in our study. Although our study revealed the expression and dysregulation of many lncRNAs in ccRCC, little is known about their roles. In addition, our understanding on the functional role of lncRNAs (e.g., *SOCS2-AS1*) is still limited. In future, further studies are needed to understand the mechanisms of how transcripts exert their function. Meanwhile, large-scale prospective studies are required to verify our findings. Furthermore, there was batch bias in the bioinformatic analysis. For instance, the *SOCS2-AS1* was not included in the common differently deregulated lncRNAs, which was down-regulated in both plasma and tissues.

## Conclusions

This study revealed differential expression patterns of lncRNAs in the plasma of ccRCC patients, involving 1,511 up-regulated and 2,153 down-regulated of lncRNAs. We also validated the expression of SOCS2-AS1 by Real-Time PCR in plasma, tissues and cell lines. In addition, we compared the differential expression level of de-regulated lncRNAs in published data and differentially expressed genes were identified. In total, 18 lncRNAs and 10 mRNAs might have diagnostic amplification for ccRCC. Ten mRNAs might have prognostic amplification.

## Data Availability Statement

All datasets presented in this study are included in the article/Supplementary Material.

## Ethics Statement

The studies involving human participants were reviewed and approved by Binzhou Medical University Hospital. The patients/participants provided their written informed consent to participate in this study.

## Author Contributions

BZ: sample collection and wrote the manuscript. HJ: data analysis and revised the manuscript. WC, LZ, LS, QS, YM, JC, and XM: sample collection and preparation of clinical materials. WC and JW: cell culture and RT-PCR. FW and JZ: RT-PCR. BH, HD, XL, and NW: data interpretation. All authors contributed to the article and approved the submitted version.

## Conflict of Interest

The authors declare that the research was conducted in the absence of any commercial or financial relationships that could be construed as a potential conflict of interest.

## Supplementary Material

Supplementary Figure 1 Significant expression of *EPB41L4B, CCND1, GGT1, CGNL1, CYSLTR1, PLAUR, UGT3A1, PROM2, MUC12*, and *PCK1* in ccRCC tissues compared to normal. Data were analyzed by ULCAN.

Supplementary Figure 2 Survival curve of *EPB41L4B, CCND1, GGT1, CGNL1, CYSLTR1, PLAUR, UGT3A1, PROM2, MUC12*, and *PCK1*. All of these genes were correlated with OS of ccRCC patients. Data were analyzed by ULCAN.
